# Haemodynamic monitoring and management in patients having noncardiac surgery: A survey among members of the European Society of Anaesthesiology and Intensive Care

**DOI:** 10.1097/EA9.0000000000000017

**Published:** 2023-01-16

**Authors:** Moritz Flick, Alexandre Joosten, Thomas W.L. Scheeren, Jacques Duranteau, Bernd Saugel

**Affiliations:** From the Department of Anesthesiology, Center of Anesthesiology and Intensive Care Medicine, University Medical Center Hamburg-Eppendorf, Hamburg, Germany (MF, BS), Department of Anaesthesiology and Intensive Care, Université Paris-Sud, Paul Brousse Hospital, Assistance Publique Hôpitaux de Paris (APHP), Villejuif, France (AJ), Department of Anaesthesiology, University Medical Centre Groningen, Groningen, the Netherlands (TWLS), Department of Anaesthesiology and Intensive Care, Assistance Publique Hôpitaux de Paris, Paris-Saclay University, Bicetre Hospital, Paris (JD) and Outcomes Research Consortium, Cleveland, Ohio, USA (BS)

## Abstract

**BACKGROUND:**

Haemodynamic monitoring and management is a mainstay of peri-operative anaesthetic care.

**OBJECTIVE:**

To determine how anaesthesiologists measure and manage blood pressure and cardiac output, and how they guide fluid administration and assess fluid responsiveness in patients having noncardiac surgery.

**DESIGN:**

Web-based survey.

**SETTING:**

Survey among members of the European Society of Anaesthesiology and Intensive Care (ESAIC) in October and November 2021.

**PARTICIPANTS:**

ESAIC members responding to the survey.

**MAIN OUTCOME MEASURES:**

Respondents’ answers to 30 questions on haemodynamic monitoring and management, and fluid therapy.

**RESULTS:**

A total of 615 fully completed surveys were analysed. Arterial catheters are usually not placed before induction of general anaesthesia (378/615; 61%) even when invasive blood pressure monitoring is planned. Mean arterial pressure (532/615; 87%) with lower intervention thresholds of 65 mmHg (183/531; 34%) or 20% below pre-operative baseline (166/531; 31%) is primarily used to guide blood pressure management. Cardiac output is most frequently measured using pulse wave analysis (548/597; 92%). However, only one-third of respondents (almost) always use cardiac output to guide haemodynamic management in high-risk patients (225/582; 39%). Dynamic cardiac preload variables are more frequently used to guide haemodynamic management than cardiac output [pulse pressure variation (almost) always: 318/589; 54%]. Standardised treatment protocols are rarely used for haemodynamic management (139/614; 23%). For fluid therapy, crystalloids are primarily used as maintenance fluids, to treat hypovolaemia, and for fluid challenges. The use of 0.9% saline and hydroxyethyl starch has declined over the last decade. The preferred methods to assess fluid responsiveness are dynamic preload variables and fluid challenges, most commonly with 250 ml of fluid (319/613; 52%).

**CONCLUSION:**

This survey provides important information how anaesthesiologists currently measure and manage blood pressure and cardiac output, and how they guide fluid administration in patients having noncardiac surgery.

**KEY POINTS**

- Arterial catheters are usually not placed before induction of general anaesthesia even when invasive blood pressure monitoring is planned.
- Mean arterial pressure with lower intervention thresholds of 65 mmHg or 20% below pre-operative baseline is primarily used to guide blood pressure management.
- Cardiac output is most frequently measured using pulse wave analysis, but only one third of respondents (almost) always use cardiac output to guide haemodynamic management in high-risk patients.
- Standardised treatment protocols are rarely used for haemodynamic management.
- The preferred methods to assess fluid responsiveness are dynamic preload variables and fluid challenges, most commonly with 250 ml of fluid.

## Introduction

Haemodynamic monitoring and management are a mainstay of peri-operative anaesthetic care. Key haemodynamic variables monitored during surgery include blood pressure and cardiac output.^1^ Interventions to maintain or optimise blood pressure and cardiac output include vasopressors, inotropes, and fluids. Fluid administration can be guided by assessing fluid responsiveness.

In 2010, a survey on haemodynamic monitoring and management conducted among members of the European Society of Anaesthesia and Intensive Care (ESAIC) and the American Society of Anesthesiologists (ASA) revealed a profound discrepancy between the growing evidence suggesting beneficial effects of intra-operative haemodynamic optimisation and its clinical implementation.^2^ Intra-operative haemodynamic monitoring and management were hardly standardised, but mostly determined by local preferences.^2^

Over the last decade, several trials have provided more evidence that optimising blood pressure^3^ and cardiac output^4–8^ can improve outcome in patients having noncardiac surgery. Additionally, new minimally and noninvasive haemodynamic monitoring methods have been developed and investigated.^9–13^ Whether new evidence and innovative technologies have changed haemodynamic monitoring and management of noncardiac surgery patients remains unknown.

In this new international survey among ESAIC members, we therefore aimed to investigate current concepts and trends in haemodynamic monitoring and management. Specifically, we aimed to determine how anaesthesiologists measure and manage blood pressure and cardiac output, and how they guide fluid administration and assess fluid responsiveness in patients having noncardiac surgery.

## Methods

We performed an anonymous web-based survey among ESAIC members using a secure web database (Survey Monkey; San Mateo, California, USA). The survey was approved by the Ethics Committee for Research in Anaesthesia and Critical Care of the French Society of Anaesthesia & Intensive Care Medicine on 27 September 2020 (IRB00010254-2020-198). The survey was endorsed by the ESAIC. Reporting of this survey adheres to the Checklist for Reporting Results of Internet E-Surveys (CHERRIES).^14^

The survey had 30 questions developed by the authors specifically for this survey; the first four were on the respondents’ work experience and setting. Some questions included a three-point Likert-scale with (almost) never, sometimes, and (almost) always, whereas others were single or multiselect answer multiple choice questions.

Eleven questions enquired about blood pressure monitoring and management during induction of anaesthesia and surgery. Seven questions asked about cardiac output monitoring and management. Some questions on cardiac output monitoring and management were based on a fictional high-risk surgical patient, specifically, a ‘72 year old male having a Whipple's procedure (pancreaticoduodenectomy) for pancreatic cancer with a history of coronary artery disease, chronic obstructive pulmonary disease and diabetes mellitus type 2’. Eight questions examined fluid administration and assessment of fluid responsiveness.

An invitation to participate that included a brief description of the survey was sent to all ESAIC members on 7 October 2021. A single reminder was sent to 4463 full members on 20 October 2021. Data collection was closed on 20 November 2021. The survey and its aims were explained in a short introduction before the first question. Participation in the survey was voluntary and no incentives were offered. Web cookies were used to allow only one response per individual. Data were collected anonymously using SurveyMonkey. We presented one to six questions on a total of 11 pages and the order of the questions remained unchanged for all participants. Consistency checks were performed automatically by the software. Participants were able to review and change their answers before submission.

We only included fully completed surveys in the analysis, but it was possible to skip individual questions. We, therefore, report the number of respondents per question. We report absolute and relative number of responses. All received responses were used as a convenience sample and no sample size calculation was performed.

Data management and analyses were conducted using Excel 2013 (Microsoft; Redmond, Washington, USA).

## Results

From a total of 728 responses, we analysed 615 (84%) fully completed surveys. Respondents’ characteristics are shown in Table 1 and Supplementary Table 1.

**Table 1:** Respondents’ characteristics

		Responses (*n* = 615)
Experience in anaesthesia, years	More than 20	209 (34%)
	11 to 20	207 (34%)
	5 to 10	139 (23%)
	Less than 5	60 (10%)
Sub-specialisation	General	263 (43%)
	Intensive care medicine	86 (14%)
	Cardiothoracic/vascular	79 (13%)
	In training/resident	38 (6%)
	Obstetric/gynaecological	26 (4%)
	Paediatric	25 (4%)
	Trauma/orthopaedic	23 (4%)
	Neuroanaesthesia	22 (4%)
	None	35 (6%)
Hospital setting	University or academic	390 (63%)
	Nonacademic <500 beds	131 (21%)
	Nonacademic >500 beds	85 (14%)
Country	Germany	87 (14%)
	Spain	35 (6%)
	Greece	33 (5%)
	Portugal	28 (5%)
	Netherlands	27 (4%)

Data are shown as absolute numbers with percentage.

### Blood pressure monitoring and management

Almost all respondents have cuff oscillometry (585/615; 95%) and arterial catheters (578/615; 94%) available for blood pressure monitoring. Finger-cuff methods for continuous noninvasive blood pressure monitoring are available to 15% of respondents (92/615).

When invasive blood pressure monitoring with an arterial catheter is planned, 61% of respondents (378/615) insert the arterial catheter during or after intubation when the patient is already under general anaesthesia. Thirty-nine percent of respondents (237/615) insert the arterial catheter before induction of general anaesthesia in awake patients.

To check the quality of the arterial blood pressure waveform and identify overdamping or underdamping, 87% of respondents (533/615) simply inspect the waveform visually. Fifty percent of respondents (311/615) perform fast-flush tests and check the dynamic response visually, whereas 8% (47/615) calculate the dynamic response. Six percent of respondents (37/615) do not routinely check the quality of the arterial blood pressure waveform at all.

**Fig. 1:**
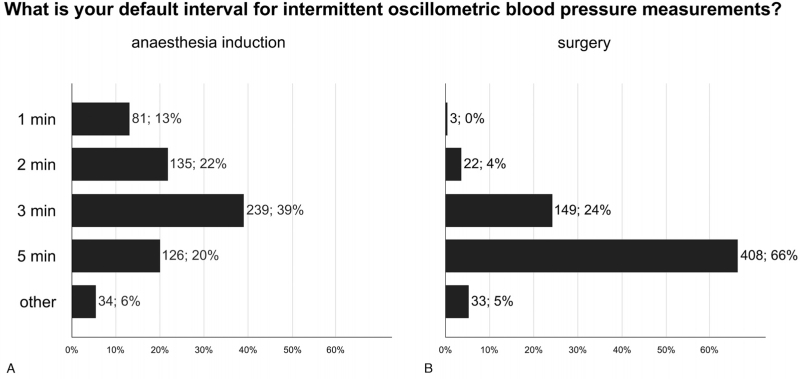
Intervals for oscillometric blood pressure measurements. Bar graphs showing the measurement intervals for oscillometric blood pressure measurements (a) during anaesthesia induction and (b) during surgery. Total number of responses 615.

When using intermittent oscillometry to measure blood pressure, the most frequently used measurement intervals are 3 min during anaesthesia induction (239/615; 39%) and 5 min during surgery (408/615; 66%) (Fig. 1).

**Fig. 2:**
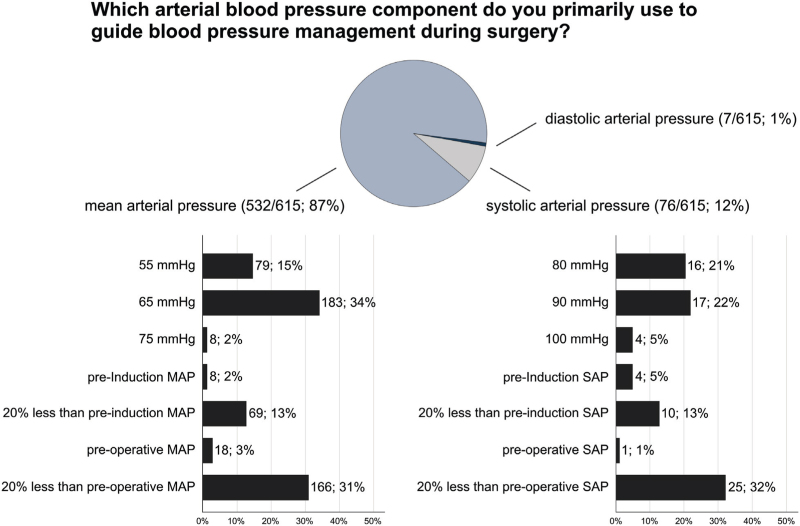
Blood pressure management. Pie chart showing the blood pressure components primarily used to guide blood pressure measurements with bar charts showing the respective blood pressure targets for mean arterial pressure (MAP) and systolic arterial pressure (SAP). Total number of responses 615.

To guide blood pressure management, 87% of respondents (532/615) primarily use mean arterial pressure, whereas 12% (76/615) use systolic arterial pressure (Fig. 2). Frequently used lower intervention thresholds for mean arterial pressure are an absolute value of 65 mmHg (183/531; 34%) and a value 20% below pre-operative baseline mean arterial pressure (from anaesthesia evaluation or from ward recordings) (166/531; 31%) (Fig. 2).

Respondents most commonly use ephedrine (219/615; 36%), norepinephrine (213/615; 35%), and phenylephrine (119/615; 19%) as primary vasopressors to maintain blood pressure (Supplement Figure 1). Seventy-four percent of respondents (458/615) perform continuous vasopressor infusion via a peripheral venous catheter in patients without a central venous catheter.

### Cardiac output monitoring and management

In the fictional high-risk surgical patient, 62% of respondents (372/597) would use pulse wave analysis to monitor cardiac output (almost) always, and 29% sometimes (176/597) (Table 2). Other cardiac output monitoring methods that would sometimes be used are transthoracic echocardiography, transoesophageal echocardiography, and transpulmonary thermodilution.

**Table 2:** Which of the following cardiac output monitoring methods would you use in this patient in your institution?

	(Almost) always	sometimes	(Almost) never
Pulse wave analysis (*n* = 597)	372 (62%)	176 (29%)	49 (8%)
Transthoracic echocardiography (*n* = 540)	37 (7%)	170 (31%)	333 (62%)
Pulse wave transit time (*n* = 540)	34 (6%)	98 (18%)	408 (76%)
Oesophageal Doppler (*n* = 539)	23 (4%)	87 (16%)	429 (80%)
Finger cuff method (*n* = 536)	21 (4%)	83 (15%)	432 (81%)
Transpulmonary thermodilution (*n* = 558)	19 (3%)	150 (27%)	386 (70%)
Transoesophageal echocardiography (*n* = 547)	15 (3%)	201 (37%)	331 (61%)
Pulmonary artery thermodilution (*n* = 558)	12 (2%)	76 (14%)	470 (84%)
Bioimpedance/bioreactance (*n* = 532)	11 (2%)	53 (10%)	468 (88%)
Lithium dilution (*n* = 533)	5 (1%)	49 (9%)	479 (90%)

Data are shown as absolute numbers with percentage.

To guide haemodynamic management in the fictional high-risk surgical patient, respondents would consider numerous advanced haemodynamic variables – most commonly pulse pressure variation [(almost) always: 318/589; 54%], cardiac output [(almost) always: 225/582; 39%], and central venous pressure [(almost) always: 208/575; 36%] (Table 3).

**Table 3:** Which of the following advanced haemodynamic variables would you use to guide haemodynamic management in this patient in your institution?

	(Almost) always	Sometimes	(Almost) never
Pulse pressure variation (*n* = 589)	318 (54%)	213 (36%)	58 (10%)
Cardiac output (*n* = 582)	225 (39%)	236 (41%)	121 (21%)
Stroke volume variation (*n* = 576)	218 (38%)	204 (35%)	150 (27%)
Central venous pressure (*n* = 575)	208 (36%)	224 (39%)	143 (25%)
Systolic pressure variation (*n* = 560)	204 (36%)	206 (37%)	150 (27%)
Central/mixed venous oxygen saturation (*n* = 580)	158 (27%)	275 (47%)	147 (25%)
Systemic vascular resistance (*n* = 559)	134 (24%)	241 (43%)	184 (33%)
Plethysmographic waveform variation (*n* = 549)	127 (23%)	219 (40%)	203 (37%)
Ejection fraction (*n* = 548)	95 (17%)	219 (40%)	234 (43%)
Venous to arterial carbon dioxide Partial pressure gap (*n* = 556)	71 (13%)	195 (35%)	290 (52%)
Global end diastolic volume (*n* = 579)	37 (7%)	167 (30%)	353 (63%)
Oxygen delivery (*n* = 562)	38 (7%)	169 (30%)	355 (63%)
Extravascular lung water (*n* = 558)	41 (7%)	187 (34%)	330 (59%)
Regional tissue oxygenation (*n* = 557)	30 (5%)	125 (22%)	402 (72%)
Pulmonary capillary wedge pressure (*n* = 556)	12 (2%)	79 (14%)	465 (84%)

Data are shown as absolute numbers with percentage.

The main reason for not using advanced haemodynamic monitoring in high-risk noncardiac surgery is that it is unavailable (329/573; 57%). Other reasons are that advanced haemodynamic monitoring is considered too expensive (165/573; 29%), too invasive (150/573; 26%), not necessary (107/573; 19%), time consuming (101/573 (18%), or not linked to distinct treatment (94/573; 16%). For 12% of respondents (68/573), unreliable measurement performance is a reason not to use advanced haemodynamic monitoring.

To treat low cardiac output, the most popular inotropes used are dobutamine (289/612; 47%), norepinephrine (155/612; 25%); epinephrine (56/612; 9%), dopamine (42/612; 7%), and ephedrine (40/612; 7%) (Supplementary Figure 2).

Twenty-three percent of respondents (139/614) use a written treatment protocol to guide haemodynamic management in high-risk noncardiac surgery patients. If a treatment protocol is available, the respondents use it mostly when there is high-patient risk or high-surgery risk (Supplementary Table 2).

Almost all respondents believe that haemodynamic monitoring and management may still improve current patient care in the operating room [(almost) always: 411/614; 67%; sometimes: 195/614; 32%].

### Fluid administration and the assessment of fluid responsiveness

Respondents’ first choice maintenance fluids are balanced crystalloid solutions (316/611; 52%) and Hartmann's or Ringer's lactate solution (246/611; 40%). 0.9% sodium chloride (0.9% saline) is used by 6% of respondents (34/611). To administer maintenance fluid, 75% of respondents (462/613) use drop infusion, 16% (95/613) use an infusion pump, and 8% (51/613) use repeated boluses.

**Fig. 3:**
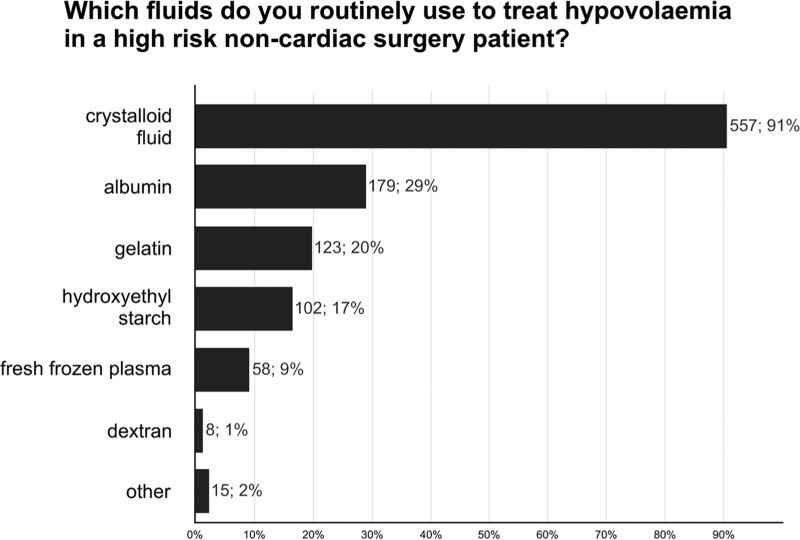
Fluids to treat hypovolaemia. Bar charts showing fluids routinely used to treat hypovolaemia in high-risk noncardiac surgery patients. Total number of responses 615.

When additional fluid administration is necessary to treat hypovolaemia in high-risk noncardiac surgery patients, respondents routinely use crystalloid fluids (557/615; 91%), albumin (179/615; 29%), gelatine (123/615; 20%), hydroxyethyl starch (102/615; 17%), and fresh frozen plasma (58/615; 9%) (Fig. 3).

Haemodynamic variables used by respondents to indicate the need for assessment of fluid responsiveness (variables triggering a fluid responsiveness test) include arterial blood pressure (455/615; 74%), lactate (408/615; 66%), urine output (394/615; 64%), pulse pressure variation (393/615; 64%), heart rate (371/615; 60%), stroke volume variation (283/615; 46%), cardiac output (274/615; 45%), mixed/central venous oxygen saturation (231/615; 38%), and central venous pressure (210/615; 34%) (Table 4).

**Table 4:** Which variables serve as an indication to assess fluid responsiveness?

	Respondents (*n* = 615)
Arterial blood pressure	455 (74%)
Lactate	408 (66%)
Urine output	394 (64%)
Pulse pressure variation	393 (64%)
Heart rate	371 (60%)
Stroke volume variation	283 (46%)
Cardiac output	274 (45%)
Central/mixed venous oxygen saturation	231 (38%)
Central venous pressure	210 (34%)
Systolic pressure variation	194 (32%)
Capillary refill time	189 (31%)
Inferior vena cava diameter/variation	175 (28%)
Plethysmographic waveform variation	148 (24%)
Global end diastolic volume	76 (12%)
No specific variable, decision to assess fluid responsiveness based on clinical experience	52 (8%)
Pulmonary capillary wedge pressure	45 (7%)
No assessment of fluid responsiveness	2 (0%)

Data are shown as absolute numbers with percentage.

Respondents most frequently assess fluid responsiveness during surgery using a standard fluid challenge of 500 ml [(almost) always: 293/599; 49%], clinical experience [(almost) always: 240/531; 45%), and dynamic cardiac preload variables [pulse pressure variation or stroke volume variation; (almost) always: 261/579; 45%].

Haemodynamic variables respondents use to diagnose whether a patient is fluid responsive or not include arterial blood pressure (417/614; 68%), pulse pressure variation (389/614; 63%), heart rate (360/614; 59%), lactate (308/614; 50%), stroke volume variation (280/614; 46%), cardiac output (256/614; 42%), systolic pressure variation (196/614; 32%), mixed/central venous oxygen saturation (183/614; 30%), and central venous pressure (176/614; 29%) (Supplementary Table 3).

**Fig. 4:**
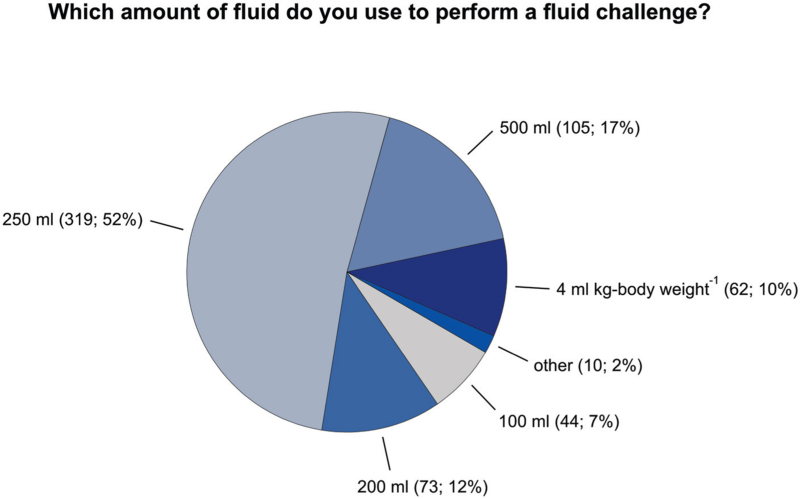
Amount of fluid for fluid challenges. Pie chart showing the amount of fluid used to perform a fluid challenge. Total number of responses 613.

First choice fluids used for fluid challenges are balanced crystalloid fluids (512/614; 83%) and 0.9% saline (70/614; 11%). Hydroxyethyl starch and other colloids are rarely used for fluid challenges (hydroxyethyl starch 7/614; 1%, albumin 5/614; 1%, gelatin 12/614; 2%). Fifty-two percent of respondents (319/613) use 250 ml of fluid for fluid challenges. Seventeen percent use 500 ml (105/613), 12% 200 ml (73/613) and 7% 100 ml (44/613); 10% (62/613) administer 4 ml kg-body weight^−1^ (Fig. 4).

## Discussion

This international web-based survey among ESAIC members provides insights into how anaesthesiologists measure and manage blood pressure and cardiac output, and how they guide fluid administration and assess fluid responsiveness.

Hypotension after induction of general anaesthesia is common and associated with organ injury.^15–17^ This survey indicates that blood pressure is usually monitored only intermittently during anaesthetic induction, with oscillometry usually set at 3 min intervals. Even when invasive blood pressure monitoring with an arterial catheter is planned, 6 out of 10 respondents of this survey will insert the catheter when the patient is anaesthetised. Clinicians presumably wait to insert arterial catheters in awake patients to avoid patient discomfort but this was not a subject of this survey. Noninvasive continuous blood pressure monitoring with finger cuff devices is now available for one out of six respondents, and may allow continuous monitoring and avoidance of hypotension during anaesthetic induction.^18^

Hypotension during surgery is also common and associated with organ injury.^19–22^ Current evidence suggests that intra-operative harm thresholds for organ injury are about 60 to 70 mmHg for mean arterial pressure and 90 to 100 mmHg for systolic arterial pressure.^23^ Almost 90% of respondents primarily use mean arterial pressure to guide blood pressure management, most often with lower intervention thresholds of 65 mmHg or 20% below preoperative baseline. However, 15% of the respondents primarily guiding blood pressure management using mean arterial pressure (13% of all respondents), still use a mean arterial pressure threshold of 55 mmHg, which is well below the population harm threshold of 60 to 70 mmHg.^22,23^

Intra-operative hypotension is routinely treated with vasopressors. The respondents of this survey prefer different vasopressors as first choice, most commonly ephedrine, norepinephrine, and phenylephrine. The choice of the vasopressor seems to depend on personal, institutional, and/or national factors (data not shown). Three-quarters of respondents infuse vasopressors via peripheral venous catheters in patients without a central venous catheter, and this is supported by research showing that adverse events from giving vasopressors via peripheral venous catheters are rare.^24,25^

In addition to blood pressure, cardiac output plays a pivotal role in the haemodynamic management of high-risk patients having noncardiac surgery. There are numerous methods to measure cardiac output.^26^ Ten years ago, only one out of three respondents in a survey among ESAIC and ASA members stated that they would monitor cardiac output in high-risk patients.^2^ In the current survey, almost 8 out of 10 respondents would monitor cardiac output at least sometimes in high-risk patients, such as the presented fictional high-risk patient. Ten years ago, two out of three respondents still used pulmonary artery thermodilution to measure cardiac output during noncardiac surgery, and only one out of three used pulse wave analysis.^2^ In contrast, this current survey shows that today pulse wave analysis is the most commonly used method for measuring cardiac output during noncardiac surgery, whereas pulmonary artery catheters are rarely used nowadays. Interestingly, the oesophageal Doppler is rarely used, although oesophageal Doppler-guided therapy may improve patient outcome.^8^ This survey also indicates that noninvasive cardiac output monitoring methods are now increasingly used even in high-risk patients.

Cardiac output-guided haemodynamic management potentially improves postoperative patient outcomes.^4,8^ However, only one out of three respondents (almost) always use cardiac output to guide haemodynamic management in high-risk noncardiac surgery patients. Dynamic cardiac preload variables are most frequently used to guide haemodynamic management by the respondents of this survey and, somewhat surprisingly, about one-third still (almost) always use central venous pressure for this purpose.

Only 23% of respondents use standardised treatment protocols for haemodynamic management, even less than the 30% of respondents using protocols in the survey 10 years ago.^2^ Obviously, optimal haemodynamic treatment strategies still need to be elucidated before widespread implementation of a distinct treatment protocol in routine care.^4,27^ Interestingly, almost 99% of our respondents think that haemodynamic monitoring and management may still improve patient care today.

Fluid management is also an important cornerstone of haemodynamic management. The survey shows that crystalloids are the first choice for maintenance fluid administration. Balanced crystalloids and Ringer's lactate (Hartmann's solution) being more often used than 0.9% saline – although there is no robust evidence to favour one over the other in surgical patients.^28,29^

Most respondents use crystalloids also to treat intra-operative hypovolaemia – and only one out of six routinely use hydroxyethyl starch. Ten years ago, one out of two ESAIC respondents still used hydroxyethyl starch to treat intra-operative hypovolaemia.^2^ The safety of hydroxyethyl starch in patients having surgery remains a matter of ongoing debate.^30^ The 2018 warnings and restrictions by the European Medicines Agency regarding the use of hydroxyethyl starch in patients having surgery may in part explain why hydroxyethyl starch today is less often used than 10 years ago.^31^

Assessing fluid responsiveness helps the guidance of fluid management.^32^ According to this survey, arterial blood pressure, dynamic cardiac preload variables, lactate, and mixed/central venous oxygen saturation trigger fluid responsiveness assessment, as does central venous pressure, although it is established that it is a poor indicator of fluid responsiveness.^33,34^ In addition, dynamic cardiac preload variables cannot reliably predict fluid responsiveness in patients with arrhythmias, spontaneous breathing activity, low tidal volume ventilation, high intra-abdominal pressure, and with an open chest.^35^ To determine whether a patient is fluid responsive or not, less than half of respondents consider cardiac output, which is surprising as fluid responsiveness is defined as an increase in stroke volume and cardiac output following fluid administration.^36^ Notably, the survey only listed ‘cardiac output’ and not ‘stroke volume’ as the suggested answer. Whether clinicians specifically consider stroke volume or cardiac output, therefore, cannot be answered by this survey. Instead of cardiac output, the respondents more commonly use arterial blood pressure, dynamic preload variables, heart rate, and lactate to determine whether a patient is fluid responsive or not.

To test fluid responsiveness, most respondents use ‘standard’ fluid challenges with 250 to 500 ml of fluid (substantially more often than mini-fluid challenges with 100 ml of fluid), dynamic preload variables, and their clinical experience. What clinical experience means for individual respondents remains unknown; it may imply that they simultaneously consider various haemodynamic variables and clinical signs. As expected, passive leg raising manoeuvres are infrequently used to assess fluid responsiveness probably because passive leg raising is impossible during surgery. To perform fluid challenges, respondents almost exclusively use crystalloids, and colloids are hardly ever used. In contrast, colloids such as hydroxyethyl starch are frequently used for fluid challenges in clinical studies.^37^

This survey was performed among ESAIC members, and the results thus primarily reflect current haemodynamic monitoring and management in Europe. Considering that the majority of our respondents were experienced, clinicians with many of them working in university or teaching hospitals, our results provide an important insight into how haemodynamic management is taught to residents. Due to the structure of the ESAIC newsletters, it is possible that individuals received the invitation to participate in the survey more than once. Therefore, the total number of invited individuals remained unclear. For the same reason, we cannot rule out that someone completed the survey more than once, but we used cookies to prevent this. Further, the questions used in the survey have not been previously used and were not externally validated before this survey.

## Conclusion

In summary, this survey provides important information how anaesthesiologists currently measure and manage blood pressure and cardiac output, and how they guide fluid administration in patients having noncardiac surgery. Arterial catheters are usually not placed before induction of general anaesthesia even when invasive blood pressure monitoring is planned. Mean arterial pressure with lower intervention thresholds of 65 mmHg or 20% below preoperative baseline is primarily used to guide blood pressure management. Cardiac output is most frequently measured using pulse wave analysis. However, only one third of respondents (almost) always use cardiac output to guide haemodynamic management in high-risk patients. Dynamic cardiac preload variables are more frequently used to guide haemodynamic management than cardiac output. Standardised treatment protocols are rarely used for haemodynamic management. For fluid therapy, crystalloids are primarily used as maintenance fluids, to treat hypovolaemia, and for fluid challenges. The use of 0.9% saline and hydroxyethyl starch has declined over the last decade. The preferred methods to assess fluid responsiveness are dynamic preload variables and fluid challenges, most commonly with 250 ml of fluid.

## Supplementary Material

Supplemental Digital Content

## Supplementary Material

Supplemental Digital Content

## Supplementary Material

Supplemental Digital Content

## Supplementary Material

Supplemental Digital Content

## Supplementary Material

Supplemental Digital Content

## References

[1] Molnar Z Benes J Saugel B . Intraoperative hypotension is just the tip of the iceberg: a call for multimodal, individualised, contextualised management of intraoperative cardiovascular dynamics. *Br J Anaesth* 2020; 125:419–423. 32690244 10.1016/j.bja.2020.05.048

[2] Cannesson M Pestel G Ricks C . Hemodynamic monitoring and management in patients undergoing high risk surgery: a survey among North American and European anesthesiologists. *Crit Care* 2011; 15:R197. 21843353 10.1186/cc10364 PMC3387639

[3] Futier E Lefrant JY Guinot PG . Effect of individualized vs standard blood pressure management strategies on postoperative organ dysfunction among high-risk patients undergoing major surgery: a randomized clinical trial. *JAMA* 2017; 318:1346–1357. 28973220 10.1001/jama.2017.14172 PMC5710560

[4] Pearse RM Harrison DA MacDonald N . Effect of a perioperative, cardiac output-guided hemodynamic therapy algorithm on outcomes following major gastrointestinal surgery: a randomized clinical trial and systematic review. *JAMA* 2014; 311:2181–2190. 24842135 10.1001/jama.2014.5305

[5] Bartha E Arfwedson C Imnell A . Randomized controlled trial of goal-directed haemodynamic treatment in patients with proximal femoral fracture. *Br J Anaesth* 2013; 110:545–553. 23274782 10.1093/bja/aes468

[6] Calvo-Vecino JM Ripollés-Melchor J Mythen MG . Effect of goal-directed haemodynamic therapy on postoperative complications in low-moderate risk surgical patients: a multicentre randomised controlled trial (FEDORA trial). *Br J Anaesth* 2018; 120:734–744. 29576114 10.1016/j.bja.2017.12.018

[7] Nicklas JY Diener O Leistenschneider M . Personalised haemodynamic management targeting baseline cardiac index in high-risk patients undergoing major abdominal surgery: a randomised single-centre clinical trial. *Br J Anaesth* 2020; 125:122–132. 32711724 10.1016/j.bja.2020.04.094

[8] Chong MA Wang Y Berbenetz NM . Does goal-directed haemodynamic and fluid therapy improve peri-operative outcomes?: A systematic review and meta-analysis. *Eur J Anaesthesiol* 2018; 35:469–483. 29369117 10.1097/EJA.0000000000000778

[9] Wagner JY Negulescu I Schofthaler M . Continuous noninvasive arterial pressure measurement using the volume clamp method: an evaluation of the CNAP device in intensive care unit patients. *J Clin Monit Comput* 2015; 29:807–813. 25726179 10.1007/s10877-015-9670-2

[10] Saugel B Hoppe P Nicklas JY . Continuous noninvasive pulse wave analysis using finger cuff technologies for arterial blood pressure and cardiac output monitoring in perioperative and intensive care medicine: a systematic review and meta-analysis. *Br J Anaesth* 2020; 125:25–37. 32475686 10.1016/j.bja.2020.03.013

[11] Saugel B Kouz K Scheeren TWL . Cardiac output estimation using pulse wave analysis-physiology, algorithms, and technologies: a narrative review. *Br J Anaesth* 2021; 126:67–76. 33246581 10.1016/j.bja.2020.09.049

[12] Kouz K Scheeren TWL de Backer D . Pulse wave analysis to estimate cardiac output. *Anesthesiology* 2021; 134:119–126. 32914174 10.1097/ALN.0000000000003553

[13] Nicklas JY Saugel B . Non-invasive hemodynamic monitoring for hemodynamic management in perioperative medicine. *Front Med* 2017; 4:209. 10.3389/fmed.2017.00209 PMC5703831 29218310

[14] Eysenbach G . Improving the quality of web surveys: the Checklist for Reporting Results of Internet E-Surveys (CHERRIES). *J Med Internet Res* 2004; 6:e34. 15471760 10.2196/jmir.6.3.e34 PMC1550605

[15] Sudfeld S Brechnitz S Wagner JY . Postinduction hypotension and early intraoperative hypotension associated with general anaesthesia. *Br J Anaesth* 2017; 119:57–64. 28974066 10.1093/bja/aex127

[16] Saugel B Bebert EJ Briesenick L . Mechanisms contributing to hypotension after anesthetic induction with sufentanil, propofol, and rocuronium: a prospective observational study. *J Clin Monit Comput* 2021; 36:341–347. 33523352 10.1007/s10877-021-00653-9 PMC9122881

[17] Maheshwari K Turan A Mao G . The association of hypotension during noncardiac surgery, before and after skin incision, with postoperative acute kidney injury: a retrospective cohort analysis. *Anaesthesia* 2018; 73:1223–1228. 30144029 10.1111/anae.14416

[18] Maheshwari K Khanna S Bajracharya GR . A randomized trial of continuous noninvasive blood pressure monitoring during noncardiac surgery. *Anesth Analg* 2018; 127:424–431. 29916861 10.1213/ANE.0000000000003482 PMC6072385

[19] Walsh M Devereaux PJ Garg AX . Relationship between intraoperative mean arterial pressure and clinical outcomes after noncardiac surgery: toward an empirical definition of hypotension. *Anesthesiology* 2013; 119:507–515. 23835589 10.1097/ALN.0b013e3182a10e26

[20] Salmasi V Maheshwari K Yang D . Relationship between intraoperative hypotension, defined by either reduction from baseline or absolute thresholds, and acute kidney and myocardial injury after noncardiac surgery: a retrospective cohort analysis. *Anesthesiology* 2017; 126:47–65. 27792044 10.1097/ALN.0000000000001432

[21] Sun LY Wijeysundera DN Tait GA . Association of intraoperative hypotension with acute kidney injury after elective noncardiac surgery. *Anesthesiology* 2015; 123:515–523. 26181335 10.1097/ALN.0000000000000765

[22] Ahuja S Mascha EJ Yang D . Associations of intraoperative radial arterial systolic, diastolic, mean, and pulse pressures with myocardial and acute kidney injury after noncardiac surgery. *Anesthesiology* 2020; 132:291–306. 31939844 10.1097/ALN.0000000000003048

[23] Saugel B Sessler DI . Perioperative blood pressure management. *Anesthesiology* 2020; 134:250–261. 10.1097/ALN.0000000000003610 33206118

[24] Pancaro C Shah N Pasma W . Risk of major complications after perioperative norepinephrine infusion through peripheral intravenous lines in a multicenter study. *Anesth Analg* 2020; 131:1060–1065. 32925324 10.1213/ANE.0000000000004445

[25] Owen VS Rosgen BK Cherak SJ . Adverse events associated with administration of vasopressor medications through a peripheral intravenous catheter: a systematic review and meta-analysis. *Meta-analysis* 2021; 25:146. 10.1186/s13054-021-03553-1 PMC8050944 33863361

[26] Saugel B Vincent J-L . Cardiac output monitoring: how to choose the optimal method for the individual patient. *Curr Opin Crit Care* 2018; 24:165–172. 29621027 10.1097/MCC.0000000000000492

[27] Kaufmann T Clement RP Scheeren TWL . Perioperative goal-directed therapy: a systematic review without meta-analysis. *Acta Anaesthesiol Scand* 2018; 62:1340–1355. 29978454 10.1111/aas.13212

[28] Maheshwari K Turan A Makarova N . Saline versus lactated Ringer's solution: the saline or Lactated Ringer's (SOLAR) Trial. *Anesthesiology* 2020; 132:614–624. 31977517 10.1097/ALN.0000000000003130

[29] Bampoe S Odor PM Dushianthan A . Perioperative administration of buffered versus nonbuffered crystalloid intravenous fluid to improve outcomes following adult surgical procedures. *Cochrane Database Syst Rev* 2017; 9:Cd004089. 28933805 10.1002/14651858.CD004089.pub3 PMC6483610

[30] Chappell D van der Linden P Ripollés-Melchor J . Safety and efficacy of tetrastarches in surgery and trauma: a systematic review and meta-analysis of randomised controlled trials. *Br J Anaesth* 2021; 127:556–568. 34330414 10.1016/j.bja.2021.06.040

[31] European Medicines Agency, Hydroxyethyl starch solutions: CMDh introduces new measures to protect patients - EMA/498908/2018 2018. Available at:www.ema.europa.eu/en/medicines/human/referrals/hydroxyethyl-starch-hes-containing-medicinal-products. [Accessed 26 January 2022]

[32] Monnet X Marik PE Teboul JL . Prediction of fluid responsiveness: an update. *Ann Intensive Care* 2016; 6:111. 27858374 10.1186/s13613-016-0216-7 PMC5114218

[33] Marik PE Cavallazzi R . Does the central venous pressure predict fluid responsiveness? An updated meta-analysis and a plea for some common sense. *Crit Care Med* 2013; 41:1774–1781. 23774337 10.1097/CCM.0b013e31828a25fd

[34] Eskesen TG Wetterslev M Perner A . Systematic review including re-analyses of 1148 individual data sets of central venous pressure as a predictor of fluid responsiveness. *Intensive Care Med* 2016; 42:324–332. 26650057 10.1007/s00134-015-4168-4

[35] Michard F Chemla D Teboul JL . Applicability of pulse pressure variation: how many shades of grey? *Crit Care* 2015; 19:144. 25887325 10.1186/s13054-015-0869-x PMC4372274

[36] Vincent J-L Cecconi M De Backer D . The fluid challenge. *Crit Care* 2020; 24:703. 33371895 10.1186/s13054-020-03443-y PMC7771055

[37] Messina A Pelaia C Bruni A . Fluid challenge during anesthesia: a systematic review and meta-analysis. *Anesth Analg* 2018; 127:1353–1364. 30300177 10.1213/ANE.0000000000003834

