# Supplementary material for: Haemodynamic monitoring and management in patients having noncardiac surgery: A survey among members of the European Society of Anaesthesiology and Intensive Care
Source: Eur J Anaesthesiol Intensive Care. 2023 Jan 16;2(1):e0017. doi: 10.1097/EA9.0000000000000017 (PMC11783660; doi:10.1097/EA9.0000000000000017)

Fig. S1. First choice vasopressor. Pie chart showing the first line vasopressors used to maintain blood pressure in non-cardiac surgery patients. *Total number of responses 615.*


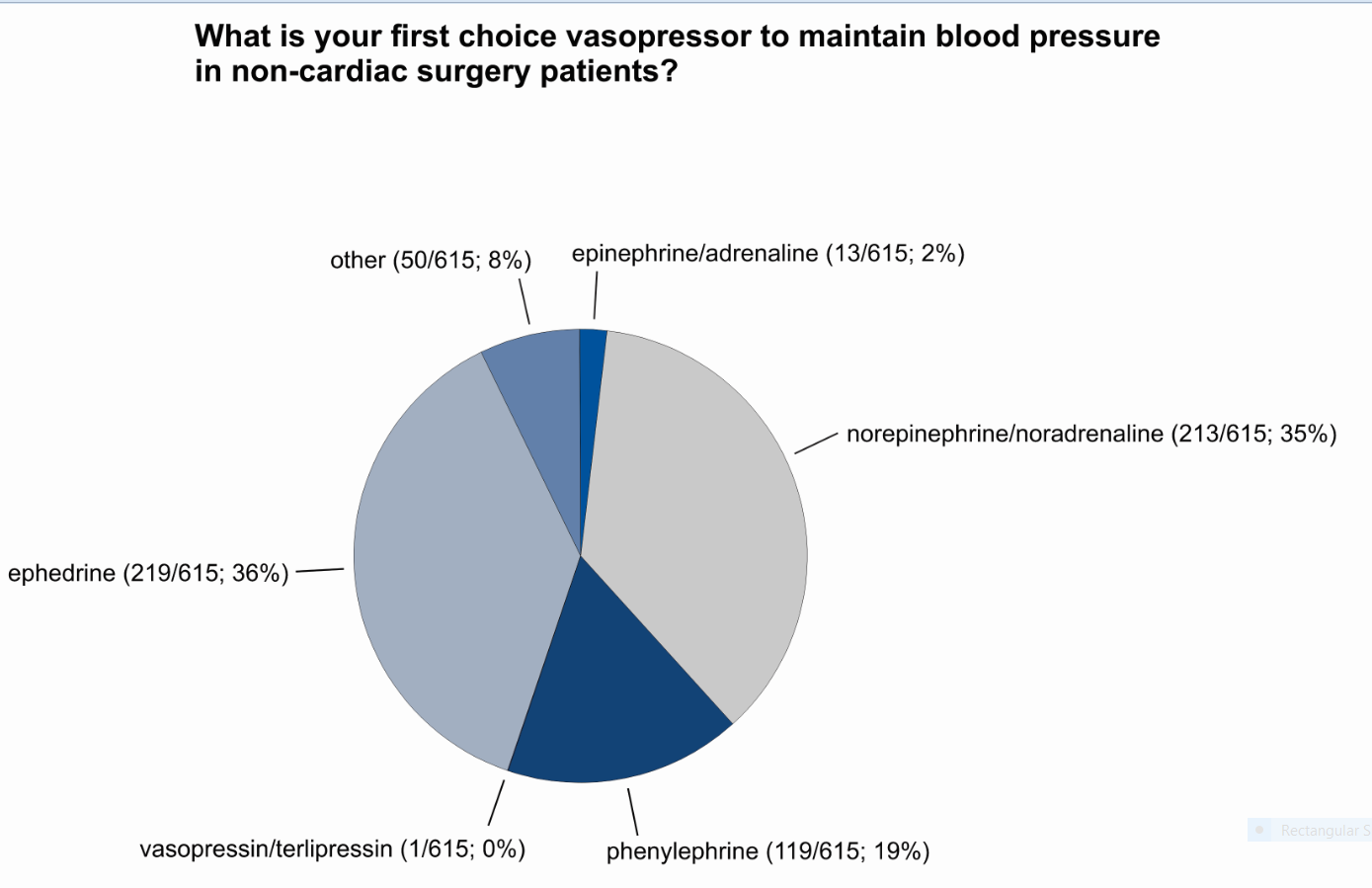

Supplement: Supplemental Digital Content [file ejaic-2-e0017-s001.doc]
