# Supplementary material for: Haemodynamic monitoring and management in patients having noncardiac surgery: A survey among members of the European Society of Anaesthesiology and Intensive Care
Source: Eur J Anaesthesiol Intensive Care. 2023 Jan 16;2(1):e0017. doi: 10.1097/EA9.0000000000000017 (PMC11783660; doi:10.1097/EA9.0000000000000017)
Supplement: Supplemental Digital Content [file ejaic-2-e0017-s003.docx]

| **Country** | **Number of Respondents (n=615)** | **Percentage of Respondents** |
| --- | --- | --- |
| Germany | 87 | 14% |
| Spain | 35 | 6% |
| Greece | 33 | 5% |
| Portugal | 28 | 5% |
| Netherlands | 27 | 4% |
| Belgium | 26 | 4% |
| Romania, Switzerland | 23 | 4% |
| Italy, United Kingdom | 22 | 4% |
| Sweden | 20 | 3% |
| Austria | 17 | 3% |
| France, India | 16 | 3% |
| Turkey | 13 | 2% |
| Norway, Russia | 12 | 2% |
| Croatia | 11 | 2% |
| Brazil, Israel | 9 | 1% |
| Poland, USA | 8 | 1% |
| Czech Republic, Saudi Arabia, Serbia | 7 | 1% |
| Denmark, Finland, Hungary | 6 | 1% |
| Australia, Bulgaria, New Zealand, Slovenia | 4 | 1% |
| Albania, Belarus, Canada, Egypt, Estonia, Ireland, South Korea, Kuwait, Latvia, Lithuania, Mongolia, Nigeria | 3 | 0% |
| Angola, Armenia, Bangladesh, Chile, Cyprus, Lebanon, Macedonia, Mexico, Moldova, Sudan, United Arab Emirates | 2 | 0% |
| Algeria, Argentina, Bahrain, Bosnia Herzegovina, China, Colombia, Democratic Republic of Congo, Grenada, Iceland, Indonesia, Iraq, Jordan, Kosovo, Malta, Namibia, Oman, Pakistan, Qatar, Singapore, Slovakia, Syria, Trinidad & Tobago, Ukraine, Uruguay, Yemen | 1 | 0% |

**Table S1. Respondents’ countries**
