# Supplementary material for: Haemodynamic monitoring and management in patients having noncardiac surgery: A survey among members of the European Society of Anaesthesiology and Intensive Care
Source: Eur J Anaesthesiol Intensive Care. 2023 Jan 16;2(1):e0017. doi: 10.1097/EA9.0000000000000017 (PMC11783660; doi:10.1097/EA9.0000000000000017)
Supplement: Supplemental Digital Content [file ejaic-2-e0017-s004.docx]

**Table S2.**

**In which non-cardiac surgery patients do you routinely use the written protocol for haemodynamic management?**

|  | Respondents (n=141) |
| --- | --- |
| Major abdominal surgery (e.g., Whipple procedure, oesophagectomy) | 105 (74%) |
| Minor abdominal surgery (e.g., cholecystectomy, hiatal hernia repair) | 9 (6%) |
| Aortic or major vascular surgery (e.g., aortic aneurysm repair, symptomatic endarterectomy) | 98 (70%) |
| Major orthopaedic surgery (e.g., hip/spine repair) | 43 (31%) |
| Minor orthopaedic surgery (e.g., shoulder/knee arthroscopy) | 5 (4%) |
| Major urologic or gynaecologic surgery (e.g., nephrectomy, prostatectomy, debulking for ovarian cancer) | 60 (43%) |
| Minor urologic or gynaecologic surgery (e.g., ureteric stenting, transurethral resection of the prostate) | 5 (4%) |
| Intracranial surgery (e.g., glioma resection) | 45 (32%) |
| Liver transplantation | 58 (41%) |
| Head or neck surgery (e.g., thyroidectomy, cochlea implant) | 18 (13%) |
| Patients with significant co-morbidities, irrespective of the surgical procedure | 90 (64%) |
| Routine use in all patients | 13 (9%) |
| No routine use, only as case-by-case decision | 23 (16%) |

This question was presented only to respondents who indicated that they use a written protocol for haemodynamic management. Data are shown as absolute numbers with percentage.
