# Supplementary material for: Haemodynamic monitoring and management in patients having noncardiac surgery: A survey among members of the European Society of Anaesthesiology and Intensive Care
Source: Eur J Anaesthesiol Intensive Care. 2023 Jan 16;2(1):e0017. doi: 10.1097/EA9.0000000000000017 (PMC11783660; doi:10.1097/EA9.0000000000000017)
Supplement: Supplemental Digital Content [file ejaic-2-e0017-s005.docx]

**Table S3.**

**Which variable do you use to assess whether the patient is fluid responsive or not?**

| Arterial blood pressure | 417 (68%) |
| --- | --- |
| Pulse pressure variation | 389 (63%) |
| Heart rate | 360 (59%) |
| Lactate | 308 (50%) |
| Stroke volume variation | 280 (46%) |
| Cardiac output | 256 (42%) |
| Systolic pressure variation | 196 (32%) |
| Central/mixed venous oxygen saturation | 183 (30%) |
| Central venous pressure | 176 (29%) |
| Capillary refill time | 144 (23%) |
| Plethysmographic waveform variation | 137 (22%) |
| Global end diastolic volume | 59 (10%) |
| No specific variable | 39 (6%) |
| Pulmonary capillary wedge pressure | 37 (6%) |
| Other | 14 (2%) |

Data is shown are absolute numbers with percentage.
